# Inflammatory recruitment of healthy hematopoietic stem and progenitor cells in the acute myeloid leukemia niche

**DOI:** 10.1038/s41375-024-02136-7

**Published:** 2024-01-16

**Authors:** Ding-Wen Chen, Jian-Meng Fan, Julie M. Schrey, Dana V. Mitchell, Seul K. Jung, Stephanie N. Hurwitz, Empar B. Perez, Mauro J. Muraro, Martin Carroll, Deanne M. Taylor, Peter Kurre

**Affiliations:** 1 Comprehensive Bone Marrow Failure Center, Division of Hematology, Department of Pediatrics, Children’s Hospital of Philadelphia, Philadelphia, PA USA; 2 Department of Biomedical and Health Informatics, Children’s Hospital of Philadelphia, Philadelphia, PA USA; 3 Single Cell Discoveries, Utrecht, Netherlands; 4 Division of Hematology/Oncology, Department of Medicine, University of Pennsylvania, Philadelphia, PA USA; 5 Perelman School of Medicine, University of Pennsylvania, Philadelphia, PA USA

**Keywords:** Cancer microenvironment, Haematopoietic stem cells

## Abstract

Inflammation in the bone marrow (BM) microenvironment is a constitutive component of leukemogenesis in acute myeloid leukemia (AML). Current evidence suggests that both leukemic blasts and stroma secrete proinflammatory factors that actively suppress the function of healthy hematopoietic stem and progenitor cells (HSPCs). HSPCs are also cellular components of the innate immune system, and we reasoned that they may actively propagate the inflammation in the leukemic niche. In two separate congenic models of AML we confirm by evaluation of the BM plasma secretome and HSPC-selective single-cell RNA sequencing (scRNA-Seq) that multipotent progenitors and long-lived stem cells adopt inflammatory gene expression programs, even at low leukemic infiltration of the BM. In particular, we observe interferon gamma (IFN-γ) pathway activation, along with secretion of its chemokine target, CXCL10. We show that AML-derived nanometer-sized extracellular vesicles (EV^AML^) are sufficient to trigger this inflammatory HSPC response, both in vitro and in vivo. Altogether, our studies indicate that HSPCs are an unrecognized component of the inflammatory adaptation of the BM by leukemic cells. The pro-inflammatory conversion and long-lived presence of HSPCs in the BM along with their regenerative re-expansion during remission may impact clonal selection and disease evolution.

## Introduction

Acute myeloid leukemia (AML) is a genetically heterogenous disease characterized by clonal expansion of myeloid blasts evolved from hematopoietic stem and progenitor cells (HSPCs) [1]. Rationally-designed, targeted therapies have led to meaningful improvements in outcome for select AML subgroups, but many patients still succumb to treatment-refractory relapse [2]. Recent studies have shown that the inflammatory secretome from AML blasts and bone marrow (BM) stroma plays a role in AML pathogenesis [3, 4]. Inflammatory signaling in the niche itself has long been linked to AML progression and hematopoietic dysfunction [5–10], and several mechanisms and mediators have been proposed including NF-κB expression programs in AML blasts [11], as well as secretion of interleukin (IL)−6 [9] and IL-1β [12]. Therefore, while AML blasts [9] and stromal cells [13] in the leukemic niche are known sources of inflammatory factors in the leukemic BM, other cellular contributors remain incompletely understood. Importantly, AML blasts are rapidly eliminated from the leukemic BM with induction therapy, and stroma cells are proportionally few, whereas HSPCs persist and re-expand in the post-remission BM. HSPCs sustain lifelong hematopoietic and immune function, adapting during homeostasis and under stress through integration of cell autonomous programs with extrinsic niche signals. The discovery that HSPCs serve as potent sensors and amplifiers of the inflammation in the BM is more recent [14], and implies a potentially durable and potent role in propagating and sustaining an inflammatory state [10].

Extracellular vesicles (EVs) generated through several biogenesis pathways are constitutively released from cells, and play a critical role for cell-cell communication in several tissues, including the BM [15]. Paracrine and endocrine trafficking of tumor derived EVs contributes to tissue adaptation and facilitates metastatic spread [16]. While EVs function in the context of conventional ligand-receptor based signaling, recent studies from our laboratory and others using xenograft models have shown that purified AML-derived extracellular vesicles (EV^AML^) are by themselves sufficient to suppress hematopoietic progenitors [3, 17], and elicit an unfolded protein response in stromal cells [18]. We also showed that long-term hematopoietic stem cells (LT-HSC) escape these suppressive effects and appear to enter a state of reversible metabolic quiescence, effected by EV^AML^ [4, 19].

To assess the inflammatory activation of endogenous, healthy HSPCs in the AML BM and overcome some of the limitations of AML xenografts, including extramedullary (splenic) hematopoiesis, we utilize two congenic murine models of AML. Results show that long-lived HSCs and MPP progenitor populations become inflammatory components of the BM niche, and that their conversion is in part mediated by EV^AML^. With the elimination of AML blasts as a source of acute inflammation, the adaptation of HSPCs gains additional relevance for its potentially long-lasting sequelae.

## Results

### HSPCs contribute to compartmental inflammation in AML niche

**Fig. 1:**
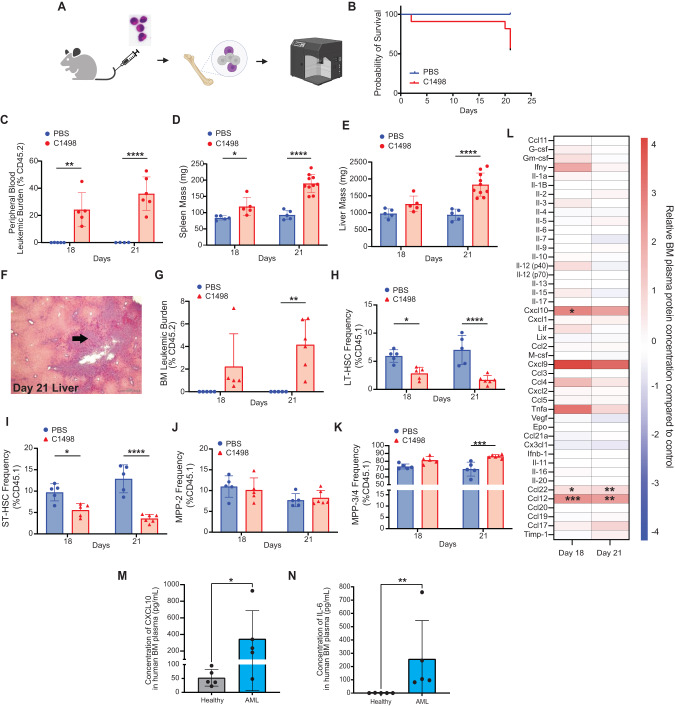
AML elicits compartmental inflammation in BM microenvironment. **A** Schematic of the experimental approach utilizing C1498 AML mouse model (*n* = 5). Endpoint analyses were performed 18- and 21-days post C1498 injection. **B** Survival probability C1498-engrafted mice compared to control. Detectable leukemic burden in PB (**C**), changes in spleen (**D**) and liver size (**E**), and evidence of extramedullary leukemic burden in liver (**F**) in C1498-engrafted mice. Detectable leukemic burden in BM (**G**), and analysis of residual HSPC subpopulation: LT-HSC (**H**), ST-HSC (**I**), MPP-2 (**J**), and MPP-3/4 (**K**) in C1498-engrafted mice. **L** Multiplex analysis of BM plasma in C1498-engrafted mice showed upregulated pro-inflammatory cytokine production compared to control. Validation studies using human BM plasma samples showed elevated pro-inflammatory cytokines, CXCL10 (**M**) and IL-6 (**N**) (*n* = 5). Values expressed as mean ± standard deviation (s.d.), Statistical significance was calculated using ANOVA. *p < 0.05; **P < 0.01; ***P < 0.001.

Inflammation has been implicated in AML pathogenesis [5–9, 13], but the inflammatory state in the AML niche more broadly, and especially at low leukemic burden, has yet to be systematically interrogated. While our prior studies and those by most other groups relied on AML xenograft approaches, we decided for the current study to investigate inflammation in the C1498 congenic murine model of AML [20–24]. Conceptually, this model enables us to evaluate the leukemic BM in the absence of potential cross-species responses [9], and avoids confounding inflammation from myeloablative conditioning [25]. First, to validate the model we intravenously (i.v.) injected C1498 AML cells (CD45.2; 1 × 10^6^ cells) into healthy non-conditioned C57BL/6 J (CD45.1) mice (Fig. 1A), with PBS-injected recipient controls. The C1498-engrafted recipients showed a median survival time around 21 days post-injection (Fig. 1B), with C1498 AML cells contributing to 24.3 ± 12.4% and 36.1 ± 12.3% of total leukocytes in the peripheral blood (PB) by day 18 and 21, respectively (Fig. 1C). PB cell count analysis at these timepoints showed a stable hematocrit, but a declining platelet count starting from day 18 (Supplementary Fig. 1A–C). Increased white blood cell counts coincided with elevated circulating leukemic blasts at both timepoints (Supplementary Fig. 1D). Necropsy examination of C1498-engrafted mice revealed splenomegaly (Fig. 1D), hepatomegaly (Fig. 1E) and a significant leukemic burden in the liver (Fig. 1F). Interestingly, despite low medullary leukemic burden (4.2 ± 2.2%; Fig. 1G), altered HSPC subpopulation frequencies were seen, including relatively reduced short-term hematopoietic stem cells (ST-HSC) and long-term hematopoietic stem cells (LT-HSC), but increased multi-potent progenitor (MPP)−3/4 cells (Fig. 1H–K). To delineate the inflammatory state of the leukemic BM at both day 18 and day 21, we utilized a multiplex Luminex platform assay to evaluate the spectrum of secreted factors in the AML niche. Here, levels of Cxcl9, Cxcl10, and Ccl12 were increased over 2-fold in BM extracellular fluid of C1498-injected mice (Fig. 1L). The data validate the C1498 model system with low medullary disease burden and recapitulate inflammation in the BM niche reported in patient studies. To assess the translational relevance of the elevated Cxcl10 observed in our murine model, we also analyzed AML patient BM plasma samples and found elevated levels of secreted CXCL10 and IL-6 compared to healthy donor BM plasma (Fig. 1M, N). Elevated IL-6 released from blasts in AML BM plasma has been previously reported [9], while elevated CXCL10 in AML BM plasma is described here for the first time. Together, the data suggest that HSPCs in the C1498 AML niche contributes to compartmental inflammation in the leukemic niche.

### HSPCs the AML niche acquire an inflammatory state

**Fig. 2:**
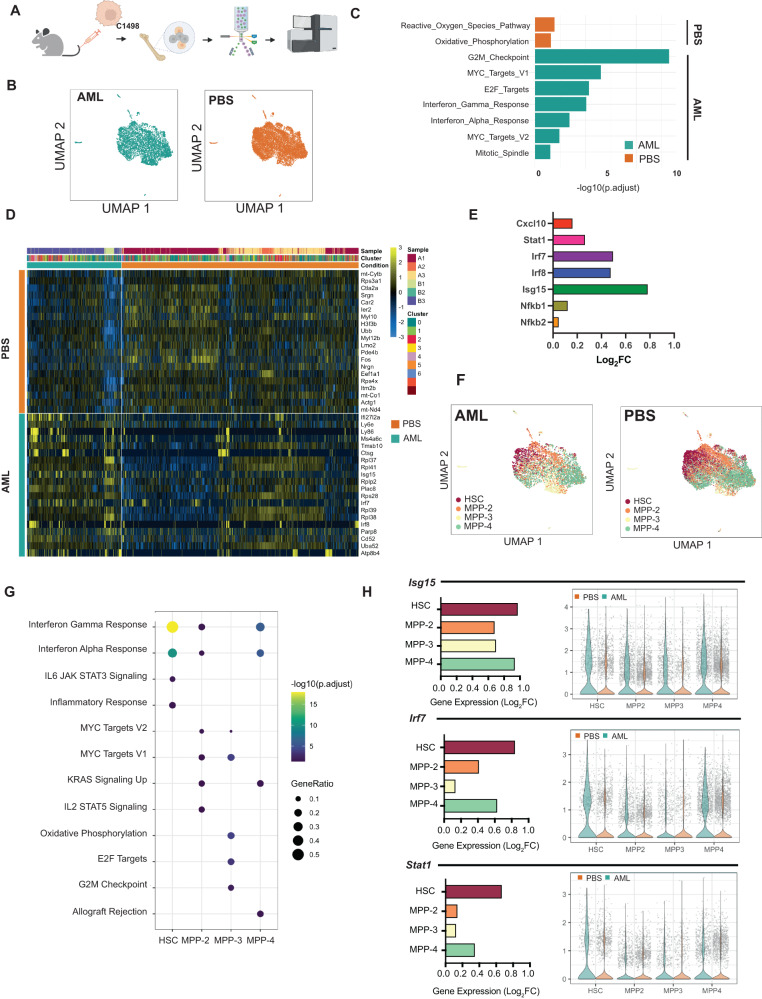
Single-cell transcriptomic analysis reveals hematopoietic stem and progenitor subpopulations exhibit an active inflammatory state in AML. **A** HSPCs from C1498-engrafted AML mice (day 21) were subjected to gene expression analysis (*n* = 3). **B** UMAP of single-cell RNA-sequenced (scRNA-Seq) HSPCs from PBS and AML mice. **C** Gene set enrichment analysis (GSEA) analysis of pathways enriched in HSPCs in AML. **D** Heatmap of top 20 differentially expressed genes by HSPCs in AML and control. **E** Gene expression of selected inflammation-related transcripts in AML. **F** UMAP of HSPC clusters defined into HSC and progenitor subpopulations (MPP-2, MPP-3, MPP-4). **G** GSEA analysis of pathways enriched in HSPC subpopulation in AML. **H** Gene expression of selected inflammation associated genes expressed in HSPC subpopulations in AML. Values expressed as mean ± s.d., Statistical significance calculated using ANOVA. *p < 0.05; **P < 0.01; ***P < 0.001.

To elucidate the potential involvement of HSPCs in inflammatory signaling in the AML niche, we evaluated fluorescent-activated cell sorting (FACS)-sorted BM HSPCs (CD45.1) at animal sacrifice using single-cell RNA sequencing (scRNA-Seq) (Fig. 2A). We first analyzed the HSPC (Lin^−^ cKit^+^ Sca1^+^) transcriptome in a pseudo-bulk approach, comparing gene expression between C1498-engrafted (HSPC^C1498^) *versus* PBS-injected (HSPC^PBS^) recipients (Fig. 2B). Over-representation analysis (ORA) showed an increase in inflammatory response related pathways (e.g. Hallmark interferon gamma and interferon alpha pathways) in HSPC^C1498^ (Fig. 2C)*.* Further examination of top differentially expressed genes (Fig. 2D) showed that several inflammatory gene targets (*Irf7, Irf8, Stat1, Nfkb1, Nfkb2, Cxcl10)* were significantly upregulated (Fig. 2E). Quantitative real-time PCR (qRT-PCR) confirmed the increased expression of several inflammation-related genes in HSPC^C1498^, including known interferon-mediated inflammatory regulator genes (i.e. *Isg15;* Supplementary Fig. 1E). Together, scRNA-Seq and qRT-PCR analysis showed that normal HSPCs in the AML BM. transcriptionally convert to a pro-inflammatory state.

To understand the potential contribution by specific stem and progenitor populations, we next classified single-cells into HSCs and multipotent progenitors (MPPs; MPP-2, MPP-3, and MPP-4) (Fig. 2F) by calculating a module score based on the expression levels of the previously described subpopulation gene signatures (Supplementary Fig. 2A) [26]. A list of differentially expressed genes in HSPC clusters included enrichment of inflammation related pathways (*Hallmark Interferon -Alpha* and *-Gamma* response pathways) in HSC, MPP-2, and MPP-4 subpopulations in AML (Fig. 2G). Analysis of top differentially expressed genes in the inflammatory response pathways reveals upregulation of several canonical inflammation response genes (*Isg15, Irf7, Stat1)* in HSCs and MPPs (Fig. 2H; Supplementary Fig. 2D, E). Gene ontology (GO) analysis of the genes differentially expressed in healthy HSC from the AML grafted cohort shows enrichment for myeloid differentiation (*p*-adjusted = 1.3 × 10^−4^) and innate immune processes (*p*-adjusted = 2.4 × 10^−5^) (Supplementary Fig. 2C). Collectively, these results demonstrate for the first time that HSCs and MPPs acquire an active inflammatory state in the AML BM.

**Fig. 3:**
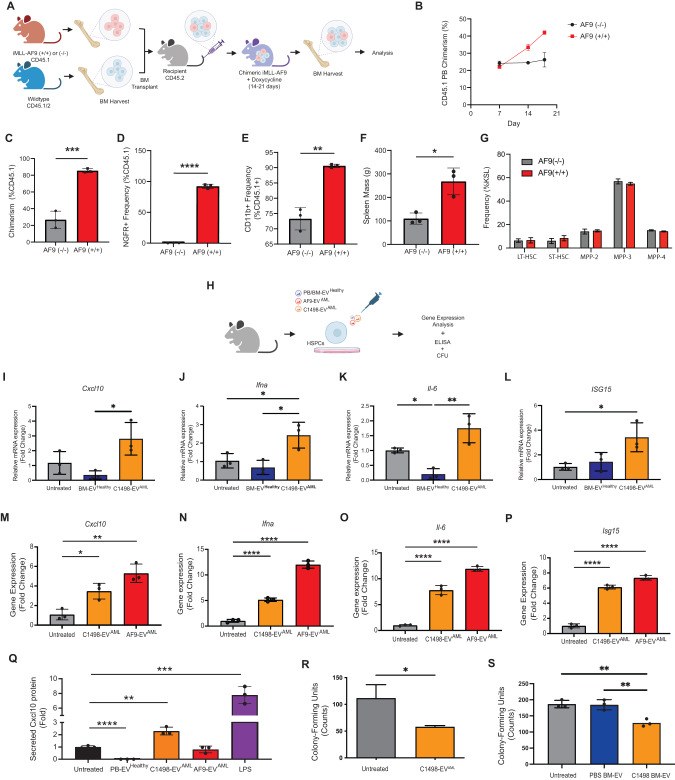
EV^AML^ incites inflammatory responses in HSPCs. **A** A schematic of iMLL-AF9 chimeric mouse model generation. **B** Assessment of leukemic hematopoietic fraction (CD45.1+) in the PB over the course of doxycycline induction. BM was assessed after 18 days of induction (*n* = 3) for leukemic fraction chimerism (**C**), the frequency of MLL-AF9-expressing cells (based on NGFR expression; cell expresses *MLL-AF9-IRES-NGFR*) (**D**), and myeloid cell frequency (**E**) in the leukemic fraction of the BM. **F** Spleen size and (**G**) LT-HSC (Lin^−^ cKit^+^ Sca1^+^ Flk2^-^ Cd150^+^ Cd48^−^), ST-HSC (Lin^−^ cKit^+^ Sca1^+^ Flk2^−^ Cd150^−^ Cd48^−^), MPP-2 (Lin^−^ cKit^+^ Sca1^+^ Flk2^−^ Cd150^+^ Cd48^+^), MPP-3 (Lin^−^ cKit^+^ Sca1^+^ Flk2^−^ Cd150^−^ Cd48^+^), MPP-4 (Lin^−^ cKit^+^ Sca1^+^ Flk2^+^ Cd150^−^ Cd48^+^) frequency were assessed (*n* = 3). **H** The role for EV^AML^ in inciting inflammatory responses in HSPCs were carried out by challenging FACS-sorted HSPCs (HSPCs sorted and pool from *n* = 3 mice per experiment) with EV^AML^. **I**, **L** Comparison of HSPC gene expression following 2 h exposure with EV derived from C1498 (C1498-EV^AML^) and healthy BM (BM-EV^Healthy^) (*n* = 3). **M**, **P** Comparison of HSPC gene expression following exposure with EV^AML^ from both C1498 and iMLL-AF9 blasts (AF9-EV^AML^) (*n* = 3). **Q** HSPC-secreted Cxcl10 were assessed 72 h following EV^AML^ exposure using ELISA. (**R**) Methylcellulose assay analysis of HSPC colony forming unit counts following 72 h challenge with either C1498-EV^AML^ (*n* = 3), or (**S**) EVs from the BM plasma of C1498-engrafted mice and PBS-injected mice (*n* = 3). Values expressed as mean ± s.d., Statistical significance calculated using ANOVA and Student’s *t*-test. *p < 0.05; **p < 0.01; ***p < 0.001; ****p < 0.0001.

To further probe for the presence of inflammation in healthy HSPCs in the AML niche, we turned to the translationally relevant MLL-AF9 mouse model of AML [27, 28]. This model system mimics human disease through doxycycline (DOX) -inducible human MLL-AF9 (iMLL-AF9) fusion oncogene expression [29, 30]. First, we generated chimeric recipients (CD45.2) bearing both iMLL-AF9 BM (CD45.1; iMLL-AF9 leukemic BM) and wildtype BM (CD45.1/2; resembling healthy BM) (Fig. 3A). Using this model, we show the emergence of a leukemic fraction in the PB following DOX induction (Fig. 3B). Assessment of BM on day 19 confirms the presence of myeloid-restricted MLL-AF9-expressing blasts (Fig. 3C–E; Supplementary Fig. 3A, B) and splenomegaly (Fig. 3F). Gene expression analysis of blasts showed upregulation of key oncogenic transcripts (hMLL-AF9, Meis1, Hoxa9) (Supplementary Fig. 3C–E). Importantly, assessment of residual normal HSPCs (CD45.1/2) showed signs of elevated inflammation (Supplementary Fig. 3F, G) without significant differences in HSPC subset distribution (Fig. 3G).

### AML-derived extracellular vesicles incite the inflammatory activation of HSPCs

We were struck by the observation that HSPCs in the C1498 AML niche are converted to an inflammatory phenotype despite low leukemic burden in the BM, and hypothesized that AML-derived extracellular vesicles (EV^AML^) may play a role [3, 4]. We first characterized the C1498 AML-derived EVs (C1498-EV^AML^) (Supplementary Fig. 4A, B) and confirmed the presence of lipid bilayer morphology vesicles with particle size of 131.0 ± 58.7 nm and enriched with known EV protein biomarkers (*Alix, Cd63, and Tsg101*) (Supplementary Fig. 4C, F). To determine whether EV^AML^ can directly incite inflammation in HSPCs, we next exposed FACS-sorted healthy HSPCs to C1498-EV^AML^ in vitro (Fig. 3H), and found transcriptional upregulation of *Cxcl10, Ifn-α, Il-6, and Isg15* (Fig. 3I–L). In contrast, EVs derived from healthy BM plasma (BM-EV^Healthy^) did not induce an inflammatory response in HSPCs. We further confirmed that EV^AML^ secreted from iMLL-AF9 blasts (AF9-EV^AML^) (Supplementary Fig. 4D, G) can also induce pro-inflammatory transcripts (*Isg15*, *Il-6*, *Ifn-α*, and *Cxcl10)* in naïve HSPC in vitro (Fig. 3M–P). Moreover, we confirmed using ELISA that HSPCs secrete significantly more Cxcl10 protein following C1498-EV^AML^ challenge (Fig. 3Q). With the observation of elevated Cxcl10 protein secretion in both the BM plasma of C1498-engrafted mice and as well in HSPC-conditioned medium following C1498-EV^AML^ exposure, our data indicate that HSPCs contribute to Cxcl10 secretion in the AML niche. Conversely, we excluded Cxcl10 secretion by C1498 cells, observing no Cxcl10 protein expression in both C1498-conditioned medium and C1498-EV^AML^ cargo (Supplementary Fig. 5A, B). Moreover, no detectable *Cxcl10* transcripts were found in C1498-EV^AML^ cargo (Supplementary Fig. 5C–D), nor was there evidence of direct mRNA transfer from EV^AML^ (Supplementary Fig. 5F, G), supporting our finding that *Cxcl10* expression is derived from EV^AML^-conditioned BM HSPCs. IFN gamma (IFN-γ) signaling pathway activation in our scRNA-Seq data [31], provides a plausible source of stimulation for *Cxcl10*. Functionally, IFN signaling and other inflammatory mediators have been shown to suppress progenitor clonogenicity and HSC stemness [14, 32]. To investigate whether inflammatory activation by C1498-EV^AML^ confers hematopoietic suppression in HSPCs, we plated primary HSPCs in methylcellulose colony-forming unit (CFU) assay following an C1498-EV^AML^ challenge ex vivo. Evaluation of the CFU colony counts confirmed reduced clonogenicity after exposure to C1498-EV^AML^ compared to control (Fig. 3R). Similarly, we observed functional progenitor suppression of HSPCs exposed to EVs derived from BM plasma of C1498-engrafted mice (BM-EV^C1498^). In contrast, HSPCs challenged with EVs derived from BM plasma of healthy controls did not exhibit clonogenic suppression (Fig. 3S). Together, our results indicate that EV^AML^ cause inflammatory activation and functionally suppress HSPC clonogenicity, albeit without a direct link to a specific the inflammatory mechanism.

To screen for potential signaling pathways involved in driving EV^AML^-mediated inflammation, we subjected HSPCs to a panel of various small molecule inhibitors of common inflammation-related signaling pathways. Mechanistically, our data suggest that EV^AML^ trigger inflammation via more than one signaling pathways. For example, pretreatment with JQ1 (1 μM), a MYC inhibitor, prior to C-1498-EV^AML^ challenge significantly suppressed *Cxcl10* and *Il-6* expression, but not *Ifna* and *Isg15 expression*, in HSPCs (Supplementary Fig. 6A top). Interestingly, in AF9-EV^AML^-challenged HSPCs pretreatment with JQ1 significantly suppressed *Ifna* and *Isg15* expression, but not *Cxcl10* and *Il6* expression, (Supplementary Fig. 6b top). Pretreatment utilizing rapamycin (1 μM) prior to C1498-EV^AML^ challenge, a mammalian target of rapamycin (mTOR) inhibitor, also significantly suppressed *Ifna* expression in HSPCs (Supplementary Fig. 6A bottom), but not in AF9-EV^AML^-challenged HSPCs (Supplementary Fig. 6B bottom). Suppression of only selected pro-inflammatory genes by JQ1 and rapamycin suggests that EV^AML^ activate HSPC through multiple pathways. Additional inhibition studies using inhibitors against other major inflammatory signaling pathways (i.e. IFNα receptor, JAK/STAT, TLR-4, NF-kB and TGF-β) failed to suppress inflammatory signaling in HSPC by C1498- (Supplementary Fig. 7) and AF9- (Supplementary Fig. 8) EV^AML^.

To gain insight into the EV^AML^ cargo responsible for regulating inflammatory responses, we assayed EVs for microRNAs (miRNA) given their known role as modulators of inflammation. miRNA profiling analysis shows that 11 inflammation-related miRNAs are enriched in both C1498- and AF9-EV^AML^ (i.e. miR-155-5p, miR-106a-5p, miR-106b-5p, miR130b-3p, miR-16-5p, miR-181a-5p, miR-19b-3p, miR-466k, miR-93-5p, miR-126a-5p) compared to healthy HSC-derived EVs (Supplementary Fig. 9A). The ensemble of candidate miRNAs present in EV^AML^ suggest that multiple pro-inflammatory signaling pathways can be potentially engaged. miR-155-5p is a master regulator of inflammation and is also found to be enriched in BM-EV derived from C1498-engrafted mice (Supplementary Fig. 9B). To investigate the involvement of miR-155 in EV^AML^, we assayed miR-155 levels in HSPCs following EV^AML^ and found elevated mR-155 levels in HSPCs at 24 and 48 h post C1498- and AF9-EV^AML^ (Supplementary Fig. 9C), respectively. Moreover, we assayed for gene expression of selected miR-155 binding targets and found significant downregulation of miR-155 targets *Msh2, Jarid2, and Wee1* following C1498- (24 h; Supplementary Fig. 9D) and AF9- (48 h; Supplementary Fig. 9E) EV^AML^ challenge, suggesting that miR-155 is involved in EV^AML^ mediated regulation but the kinetics differ between C1498- and AF9- EV^AML^. In summary, our data shows that EV^AML^ carry a proinflammatory miRNA cargo and incite inflammation in HSPCs via multiple signaling pathways.

### EV^AML^ incite inflammatory responses in HSPCs in vivo

**Fig. 4:**
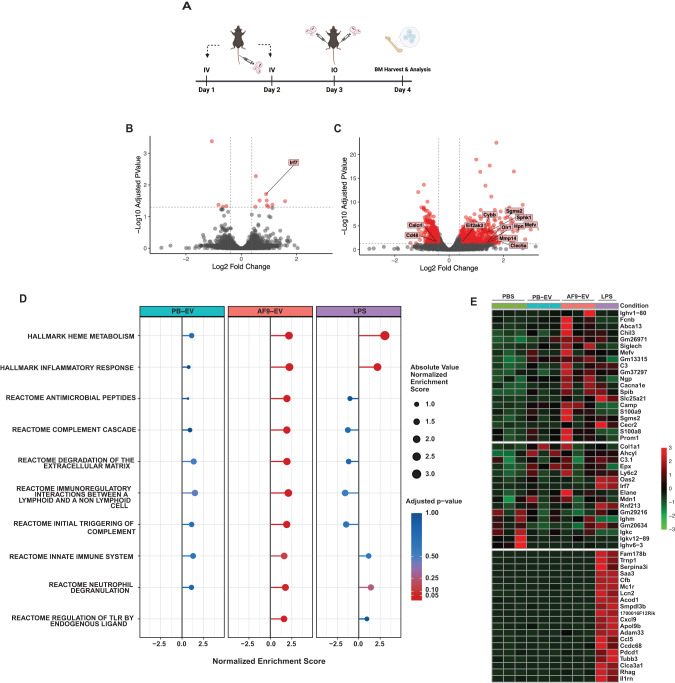
EV^AML^ incites inflammatory responses in HSPCs in vivo. **A** B6 CD45.1 mice were serially injected with AF9-EV^AML^ (2E9 particles/day) for 3 days (*n* = 3). Mice received either PBS or PB-EV (2E9 particles/day) served as negative controls. HSPCs from injected mice were harvested 24 h the last injection and subjected to RNA-Seq analysis. Volcano plots illustrating differential HSPCs gene expression between: (**B**) PB-EV and (**C**) AF9-EV^AML^. **D** GSEA analysis of differential expressed genes in HSPCs from AF9-EV^AML^ showed enriched inflammatory related pathways compared to vehicle control (PBS-injected). **E** Heatmap of top 20 inflammatory targets significantly upregulated in HSPCs from AF9-EV^AML^- (top), PB-EV- (mid), and LPS- injected (lower panel) mice compared to vehicle control (PBS-injected).

To determine whether EV^AML^ can incite inflammatory signaling in HSPCs in vivo, we subjected healthy C57BL/6 J mice to serial administration (once daily for 3 days) of EV^AML^ (Fig. 4A). Transcriptomic analysis of HSPCs (KSL) from AF9-EV^AML^-injected mice (HSPC^AF9-EV^) harbored a distinctly different transcriptome profile compared to HSPCs from PBS- and healthy peripheral blood (PB)-EV- injected recipients (Fig. 4B, C**;** Supplementary Fig. 4E, H). Notably, gene set enrichment analysis (GSEA) revealed enrichment of several inflammatory- and immune response related pathways (Fig. 4D). Along with upregulation of several well annotated inflammation related genes (*S100a8*, *S100a9, Sphk1, Mmp14, Clec5a, Sgms2s)* in HSPC^AF9-EV^ (Fig. 4E), these results indicate that AF9-EV^AML^ can by themselves induce innate immune responses in vivo. Prior studies have reported upregulation of Sca-1 surface protein expression in non-HSPC progenitors (Lin^−^ cKit^+^ Sca1^−^) under inflammatory stress, which can impact conventional HSPC (Lin^−^ cKit^+^ Sca1^+^) immunophenotyping and FACS sorting [33]. To exclude this confounder, we compared HSPC subsets assignment using conventional LSK with an alternate immunophenotyping strategy: LSK (Lin^−^ cKit^+^ Sca1^+^; Supplementary Fig. 10A) and L86K (Lin^−^ CD86^+^ cKit^+^; Supplementary Fig. 10B). These results confirmed that, in contrast with the well described increase in LSK frequency following LPS challenge, there were no significant differences between LSK and L86K frequencies following AF9-EV^AML^ challenge compared to controls (Supplementary Fig. 10C–I). Altogether, our results show AF9-EV^AML^ can elicit the inflammatory conversion in HSPCs.

### Human AML-derived EVs trigger inflammatory programs in human CD34 HSPCs

**Fig. 5:**
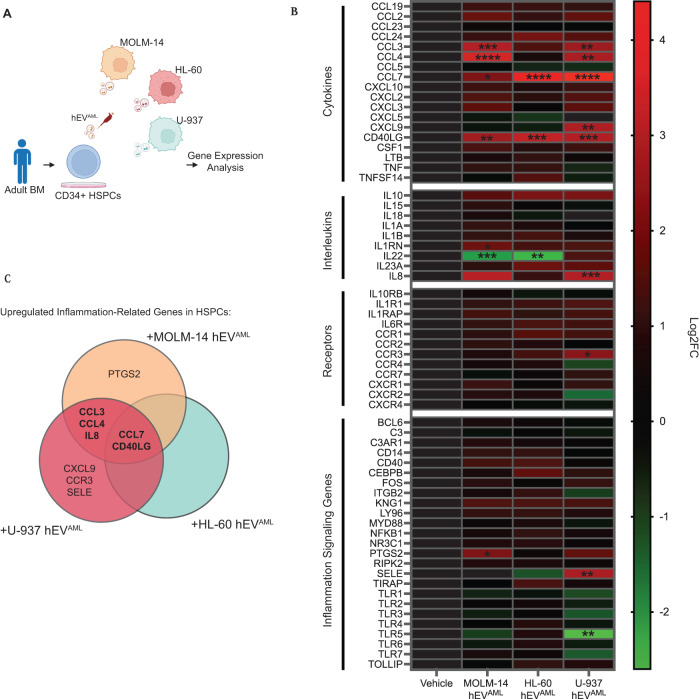
Inflammatory signaling in human CD34 HSPCs following human AML-derived EV^AML^ exposure. **A** Schematic of hEV^AML^ challenge on BM CD34 cells ex vivo. **B** Averaged gene expression analysis of CD34 cells following MOLM-14- (*n* = 4), HL-60- (*n* = 4), and U-937-(*n* = 4) hEV^AML^. **C** Venn diagram of the inflammatory gene expression profile in HSPCs challenged with different hEV^AML^. Statistical significance calculated using ANOVA. *p < 0.05; **p < 0.01; ***p < 0.001; ****p < 0.0001.

To assess the translational significance of our findings, we asked whether human AML-derived EVs (hEV^AML^) can also incite inflammatory activation in human HSPCs. To test this, we harvested hEV^AML^ (MOLM-14-, HL-60-, and U-937-hEV^AML^) derived from three different human AML cell lines (MOLM14, HL-60, and U-937) and subjected BM hCD34 cells to hEV^AML^ exposure challenge (Fig. 5A). Gene expression analysis of hCD34 reveals that MOLM-14-, HL-60-, and U-937-hEV^AML^ incited inflammatory activation in hCD34 cells (Fig. 5B). Interestingly, all three hEV^AML^ triggered significantly elevated expression of CCL7 and CD40LG in hCD34 HSPCs. In addition, MOLM-14- and U-937- hEV^AML^ also triggered upregulated expression of CCL3, CCL4, and IL8 (Fig. 5C). Altogether, the results herein reinforce our findings that EV^AML^ plays a role in triggering inflammatory activation in HSPCs in human.

## Discussion

Inflammation has long been associated with AML pathogenesis, accounting for the suppression of healthy HSPCs, leukemia promotion and clonal selection [34]. Yet, comprehensive insight into the cellular sources, mediators and mechanisms remains elusive, in part constrained by the reliance on experimental modeling in xenografts. We hypothesized that healthy HSPC are more than simple bystanders and play an active role in the inflammatory leukemic niche. The resulting studies in two congenic murine models of AML now reveal for the first time the extent to which healthy HSPC subpopulations undergo pro-inflammatory conversion in the leukemic niche. We show that this process involves EV^AML^ crosstalk that activates interferon pathways and upregulates expression of the pro-inflammatory chemokine *Cxcl10*, among others, in healthy HSPCs.

Prior studies of AML link inflammatory cytokine secretion to AML blasts and BM stromal components [6, 12]. Unlike reports in myeloproliferative neoplasms, the role of residual healthy hematopoietic cells in AML and their contribution to the inflammatory secretome has received relatively little attention [35, 36]. Here, we dissected the role of immunophenotypic HSPC and demonstrate that the single cell transcriptome aligns with the BM plasma secretome even at low leukemic burden. Work in both C1498 and iMLL-AF9 AML models consistently demonstrates that HSPCs undergo an inflammatory conversion in the AML niche that prominently involves interferon signaling [37]. GSEA and GO analysis of HSCs in AML reveal pathway enrichment for myeloid differentiation and innate immune response processes, previously reported in models of experimental LPS-induced inflammation in HSPC [38]. Intriguingly, immunophenotypically defined HSCs show robust *Cxcl9* and *Cxcl10* gene expression, both targets of IFN-γ pathway activation, when neither is secreted by AML blasts. Perhaps not surprisingly, given their diverse cargo, our results suggest that EV^AML^ can trigger multiple inflammatory signaling axes (including mTOR and MYC associated pathways) in HSPCs, precluding the easy identification of a unifying mechanism in our studies. Our examination of miRNA content in EV^AM^ may provide the strongest lead in its repertoire of inflammation-related miRNA cargo. In addition to the potential involvement of miR-155, the enrichment of miR-181b-5p in AF9-EV^AML^ also offers direct connection to our prior study of uniquely enriched small RNA in AML patient plasma EVs [39].

Our studies further revise the view of HSPCs as passive bystanders that are physically displaced from niche occupancy by encroaching AML blasts. Several groups showed that active crosstalk by leukemic cells at low tumor burden can elicit progenitor suppression while preserving long-lived HSCs in the BM [40–44]. However, while these changes are reversible, adaptation to sustained inflammatory stress may impose durable changes in HSC with potential generation of preleukemic clones, [42, 45], or as a source of selective pressure that promotes clonal amplification [46, 47]. For example, clonal hematopoiesis with indeterminate potential (CHIP), more commonly in AML patients, is one of the conditions closely associated with inflammation, wherein clonal expansion compromises long-term HSC function [48]. Inflammation significantly alters fate and function of HSC through regulation of survival factors, myelopoiesis and immunosuppressive response [38], but clonal restriction through selective expansion of clones bearing CHIP mutations promotes systemic inflammatory sequelae [49].

Adding HSPC to the list of cellular sources of the inflammatory secretome, the current observations expand our understanding of the AML BM as a self-reinforcing inflammatory niche and potential sanctuary for disease persistence [12]. HSPC secreted factors, such as CXCL10 have not been evaluated in the setting of drug resistance in AML, but have been suggested to promote chemoresistance [50] and blast migration to the BM of an acute lymphoblastic leukemia model [51]. Other studies showed that CXCL10 suppresses colony formation by human hematopoietic progenitors [52].

Our results of hEV^AML^ exposure of healthy hCD34 cells not only extend our mouse model findings, but also provide insight into how hEV^AML^ derived from different AML subtypes (FAB Types M2, M5, and M5a) can exert both shared and unique inflammatory stimuli on HSPCs. In terms of similarities, all three hEV^AML^ incited immune cell chemotaxins CCL7 and CD40LG expression in CD34 HSPCs. Notably, CD40L can serve a pro-leukemic role by promoting proliferation and anti-apoptotic effects in AML blasts [53], and boost inflammatory cytokine secretion (e.g. IL-6) in AML blasts [53]. A comparison between the two monocytic leukemia cell lines, MOLM-14 (FAB type M5) and U-937 (FAB type M5a), further revealed similarities amongst the monocytic leukemias as they both also incited upregulation of CCL3, CCL4 and IL-8 in HSPCs, which were not observed in HL-60 (promyelocytic leukemia; FAB type M2). All three cytokines have been associated with poor prognosis in AML [54–56].

The translational significance of an inflammation-primed HSPC compartment also informs our understanding of the delayed hematopoietic recovery of cytopenic patients [14]. Moreover, HSPCs may serve as long-lived cytokine producers, potentially contributing to drug resistance and relapse in AML [56–58]. While our study provides unambiguous evidence for inflammatory adaptation of HSPC in the leukemic BM, we cannot comment on the long-term consequences and durability. We also cannot comment on the EV specific mechanism that elicits inflammation, and the data do not fully exclude components of the non-EV secretome from the observed effects. We anticipate that inflammation will vary depending on AML subtype and age of the patient. Clinically, a recent study in pediatric and adult AML patients showed that inflammation is present at any stage of AML, independent of tumor burden [9], correlating in another study with worse overall survival and led to the proposal of a new scoring system that incorporates inflammation into an outcome risk score [59]. Broadly, modulating inflammation [60] may offer significant benefits in AML patients beyond remission induction.

Altogether, our report provides first evidence that healthy HSPC contribute to the compartmental inflammation in the AML niche. Such a conversion can be elicited by EV trafficking, leaving HSPCs as long-lived hubs of inflammatory activity with a potentially profound impact subsequent response to infection or re-emergence of disease.

## Methods

Due to space constraints, please refer to the “Supplementary – Expanded Methods” section for extensive methods description.

### Supplementary information

Supplementary Figures and Tables

Supplementary Expanded Methods

## Data Availability

Raw sequencing data are deposited into Gene Expression Omnibus (GEO) database and accessible by the public. 1) Bulk RNA-Seq gene expression dataset of HSPCs following AF9-EV^AML^ (Accession ID: GSE239307); 2) ScRNA-Seq gene expression dataset of HSPCs in C1498-engrafted mice (Accession ID: GSE238091).
