# Supplementary material for: Inflammatory recruitment of healthy hematopoietic stem and progenitor cells in the acute myeloid leukemia niche
Source: Leukemia. 2024 Jan 16;38(4):741–50. doi: 10.1038/s41375-024-02136-7 (PMC10997516; doi:10.1038/s41375-024-02136-7)
Supplement: Supplementary file 1 — Supplementary Figures and Tables [file 41375_2024_2136_MOESM1_ESM.pdf]

Supplementary Figure 1

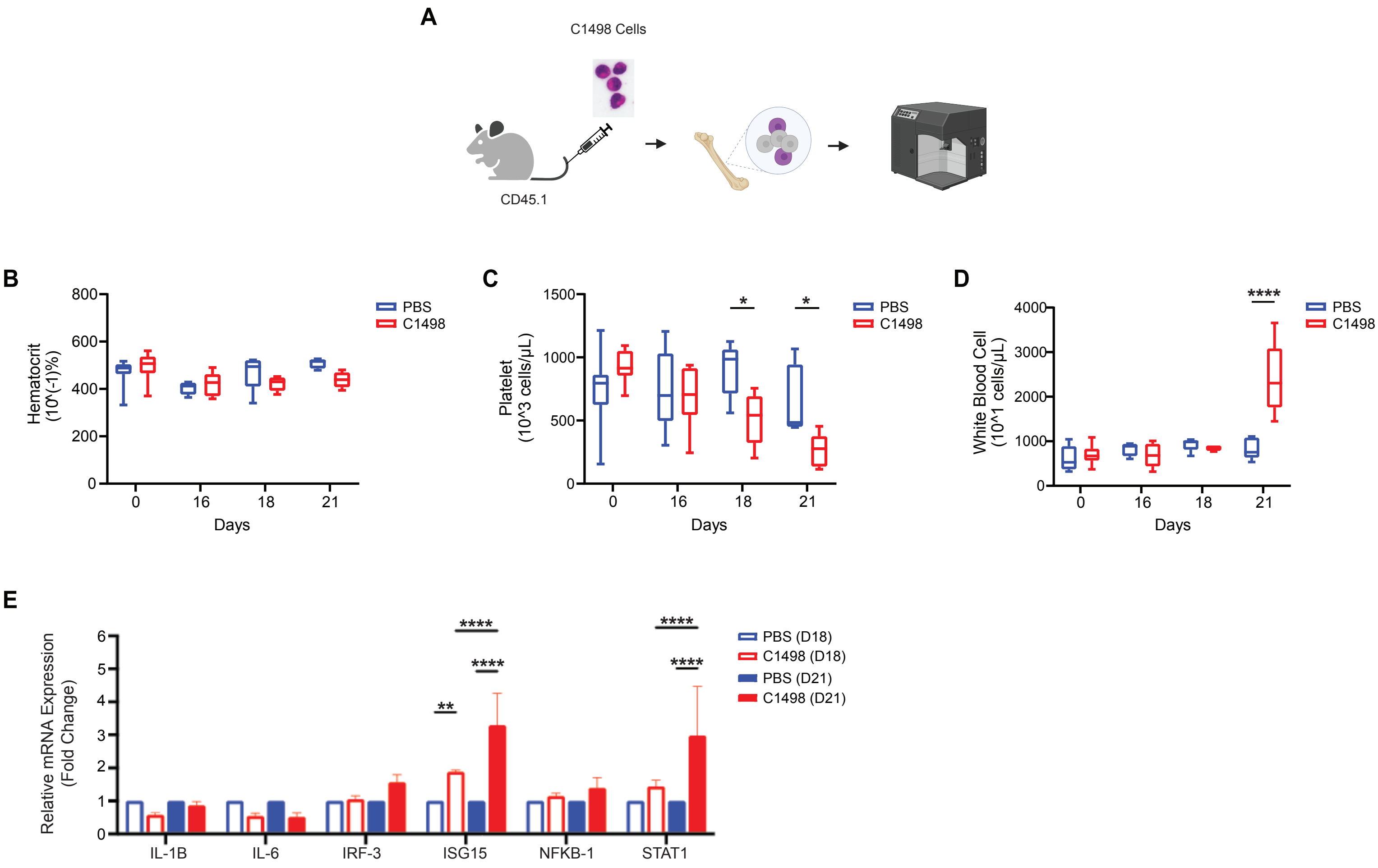

Supplementary Figure 1 – Additional characterization data relating to C1498 model. Complete blood count analysing showing hematocrit (B), platelet (C), white blood cell (D) counts. Quantiative real-time PCR analysis of HSPCs (Lin- cKit+ Sca1+) gene expression 18 and 21 days post C1498 engraftment. Values expressed as mean±s.d., Statistical significance calculated using ANOVA. * p<0.05; ** p<0.01; *** p<0.001; **** p<0.0001.

Supplementary Figure 2

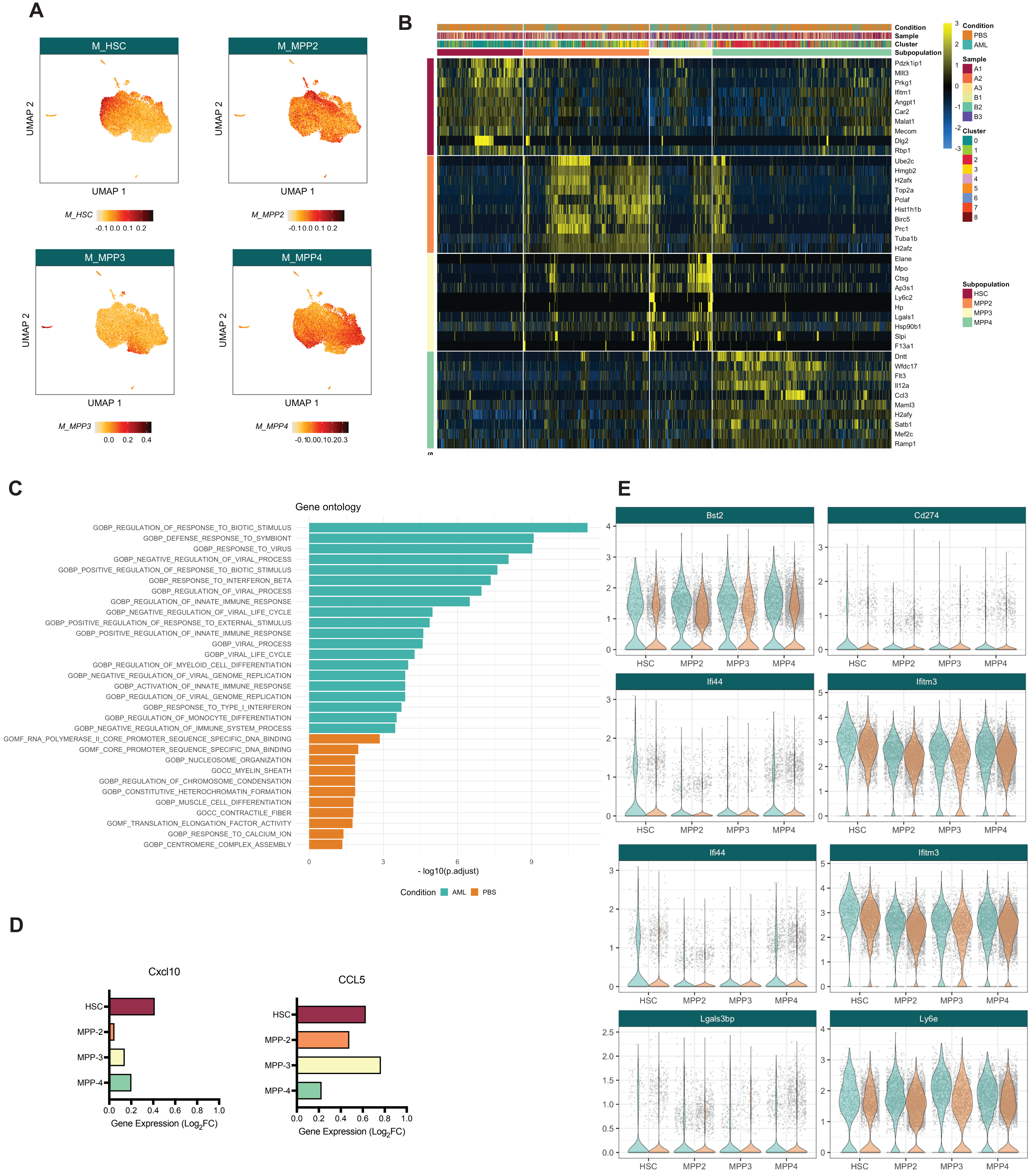

Supplementary Figure 2 – (A) UMAP showing module score of HSC, MPP-2, MPP-3, and MPP-4 gene signatures. (B) Top differentially expressed genes between HSC, MPP-2, MPP-3, and MPP-4 subpopulations. (C) Gene ontology (GO) analysis of enriched processes in HSCs. (D) Selected inflammatory cytokine encoding genes and, (E) leading edge genes from Interferon Response Pathways upregulated in HSC and MPPs in AML.

Supplementary Figure 3

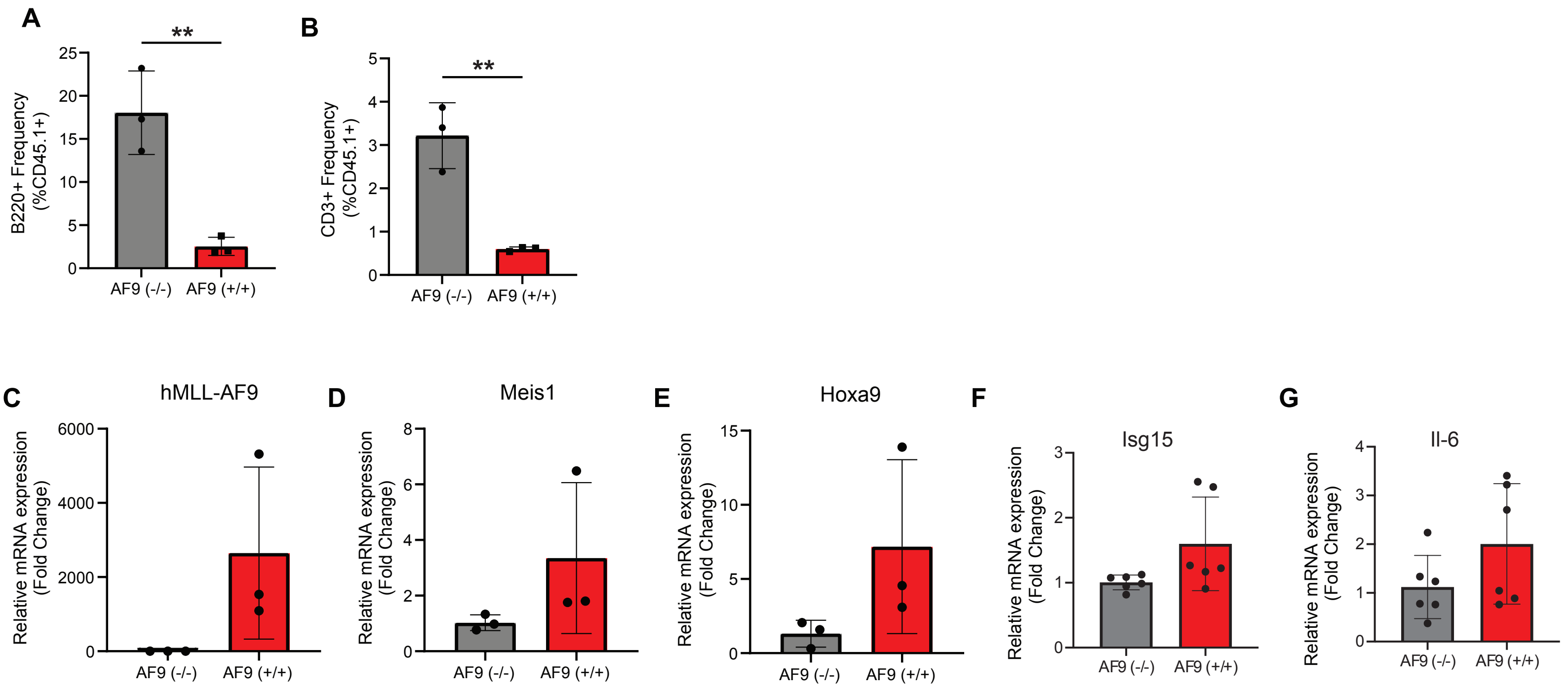

Supplementary Figure 3 – Reduced BM B220+ CD3+ population subsets following DOX-induction in iMLL-AF9 mice (+/+) carrying homozygous MLL-AF9 allele (+/+), compared to control mice without MLL-AF9 allele (-/-) (A-B). Gene expression analysis of AF9 blasts (CD45.1+; C-D) and normal HSPCs (CD45.1/2+; E-G). Values expressed as mean±s.d., Statistical significance calculated using Student's t-test. * $p<0.05$; ** $p<0.01$; *** $p<0.001$; **** $p<0.0001$.

Supplementary Figure 4

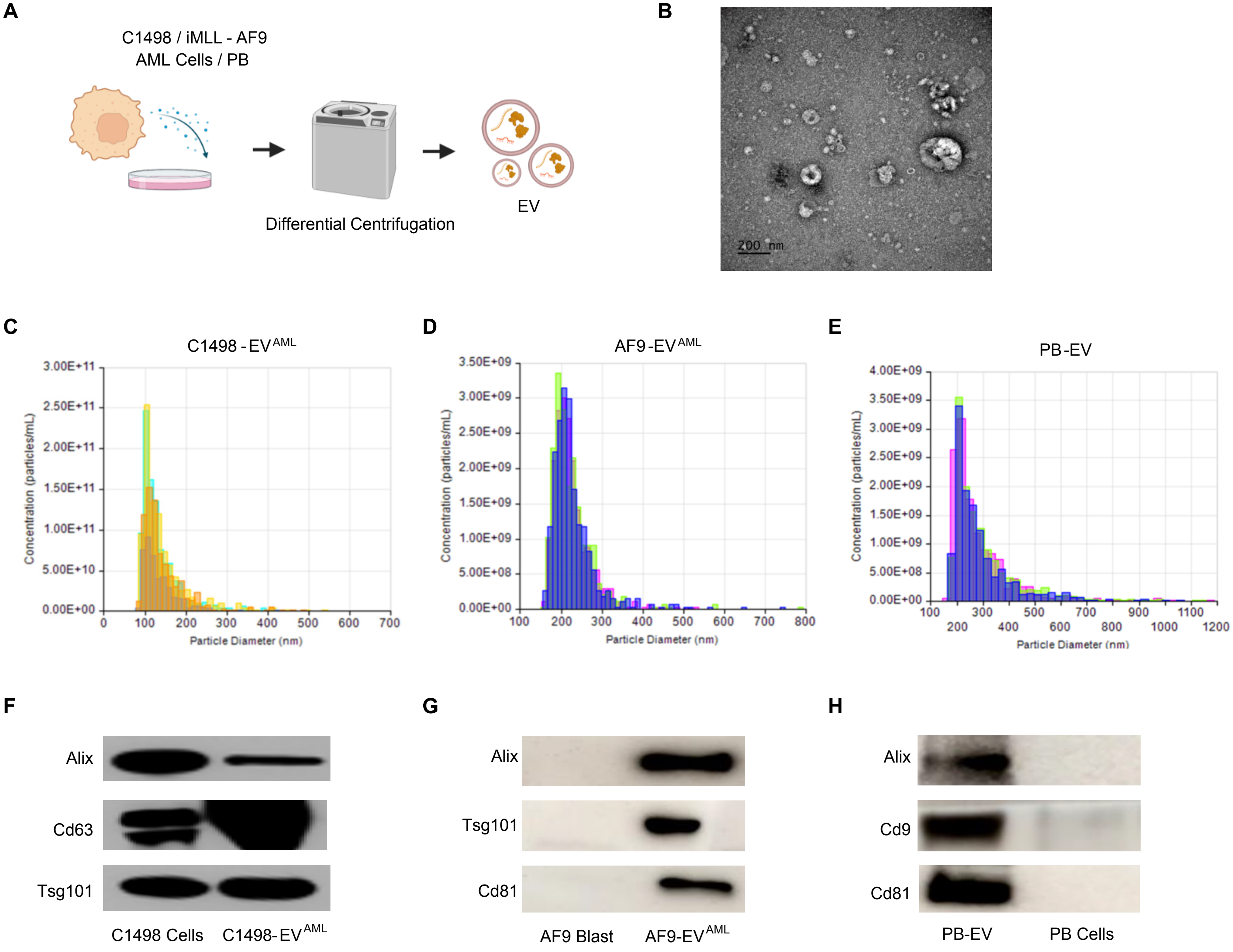

Supplementary Figure 4 – Extracellular vesicle harvest schematic (A). Transmission electron microscopy image of C1498-EV^{AML} (B). EV particle quantitation and biomarker characterization for C1498-EV^{AML}(C,F), AF9-EV^{AML}(D,G), PB-EV^{Healthy} (E,H),

Supplementary Figure 5

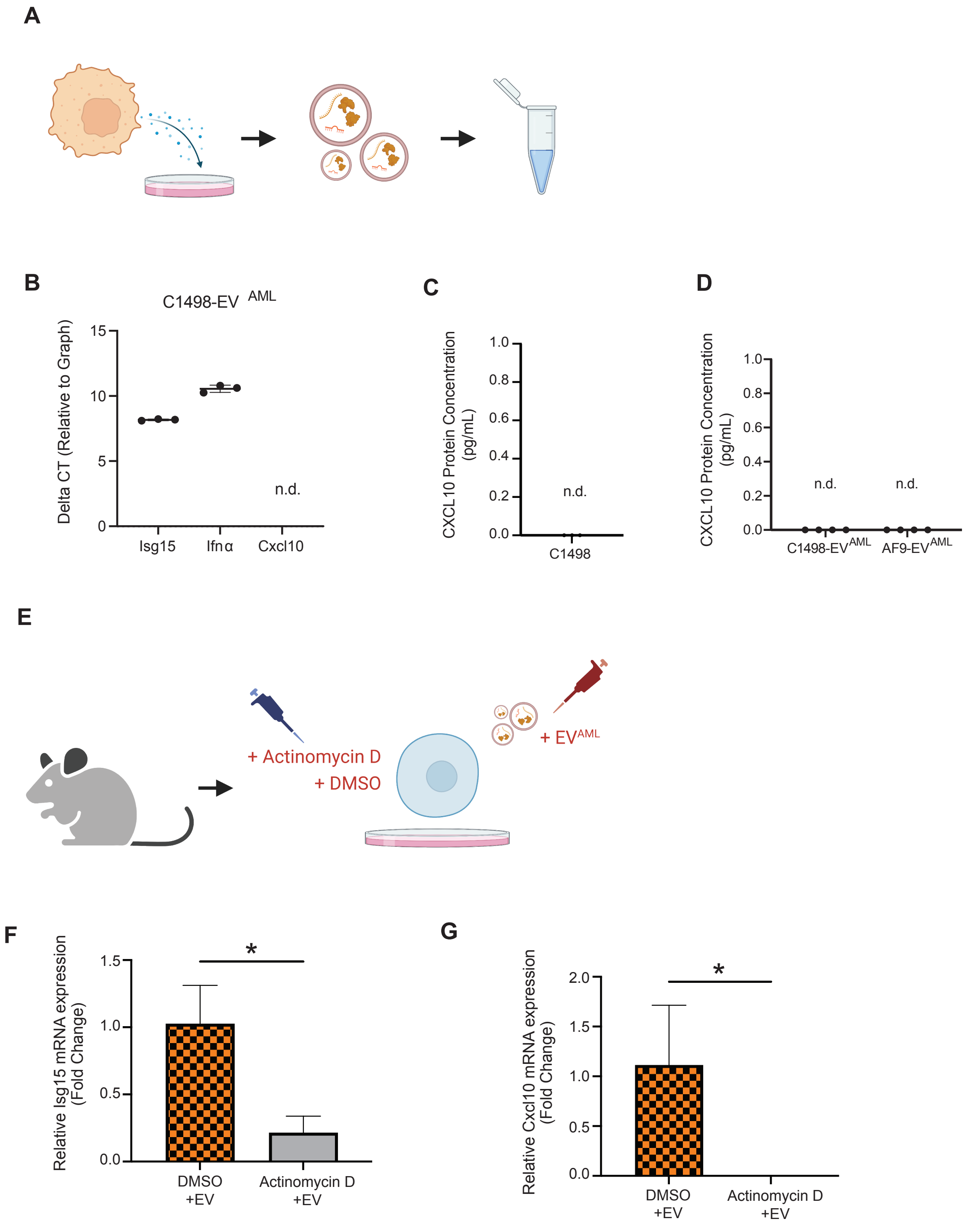

Supplementary Figure 5 - Schematic of RNA analysis in EVs (A) and relative delta CT of target RNA compared to GAPDH (B). Cxcl10 proteins were not detected (n.d.) in C1498-conditioned medium (C) and EV^{AML} (D). Schematic of Actinomycin D treatment experiment (E), and the gene expression analysis of HSPCs following EV challenge, with or without Actinomycin D pretreatment (F-G). Values expressed as mean±s.d., Statistical significance calculated using Student's t-test. * $p<0.05$. n.d. represent not detected.

Supplementary Figure 6

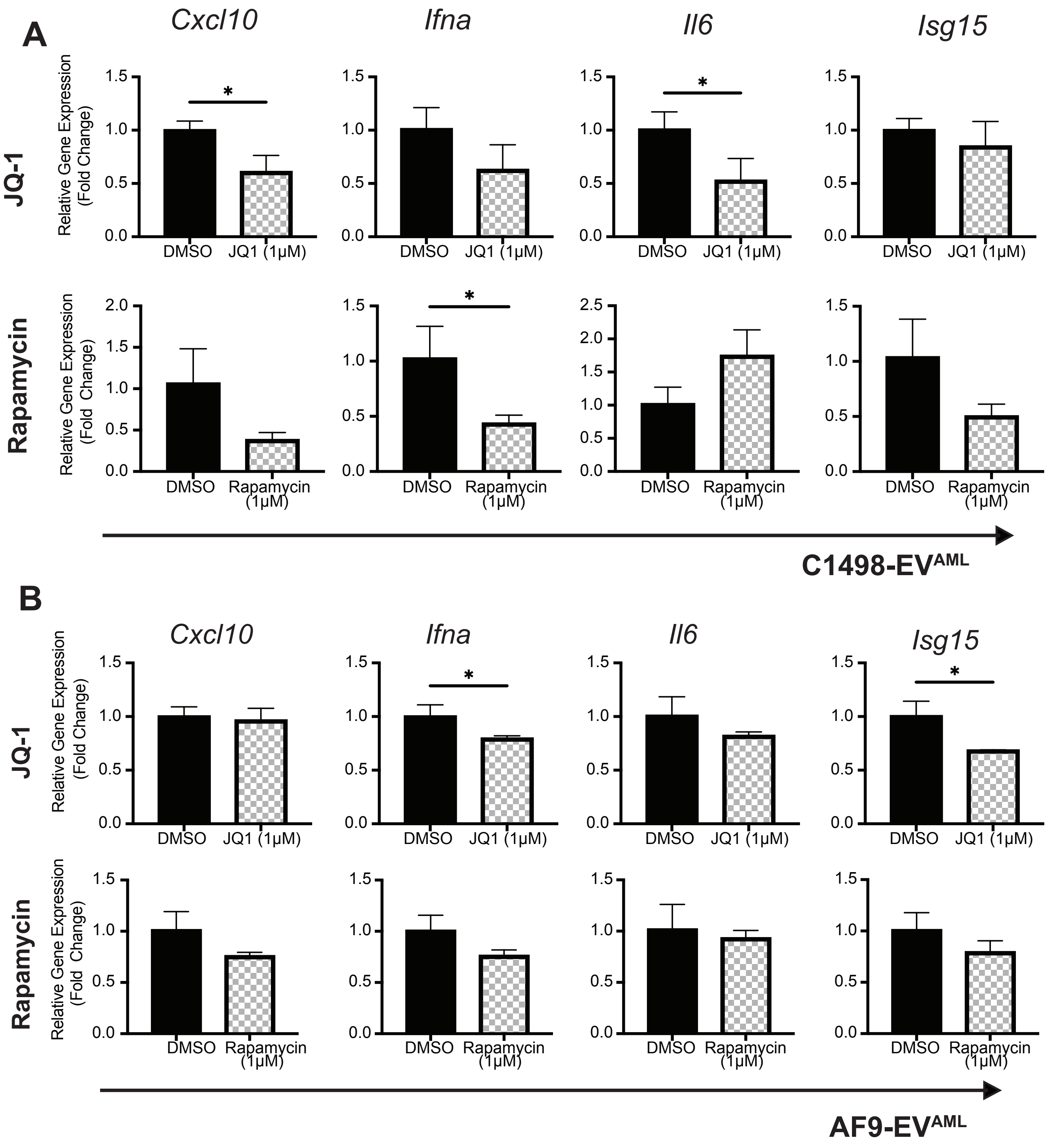

Supplemenatry Figure 6 - Multiple pathways are involved in EVAML-mediated inflammatory responses in HSPCs. Differential gene expression analysis in FACS-sorted HSPCs pretreated with small molecule inhibitors JQ-1 (1μM), rapamycin (1μM) for 3 hours prior to (A) C1498-EV^{AML} and (B) AF9-EV^{AML} challenge (n=3). Values expressed as mean±s.d., Statistical significance calculated using Stduent's t-test. * $p<0.05$.

Supplementary Figure 7

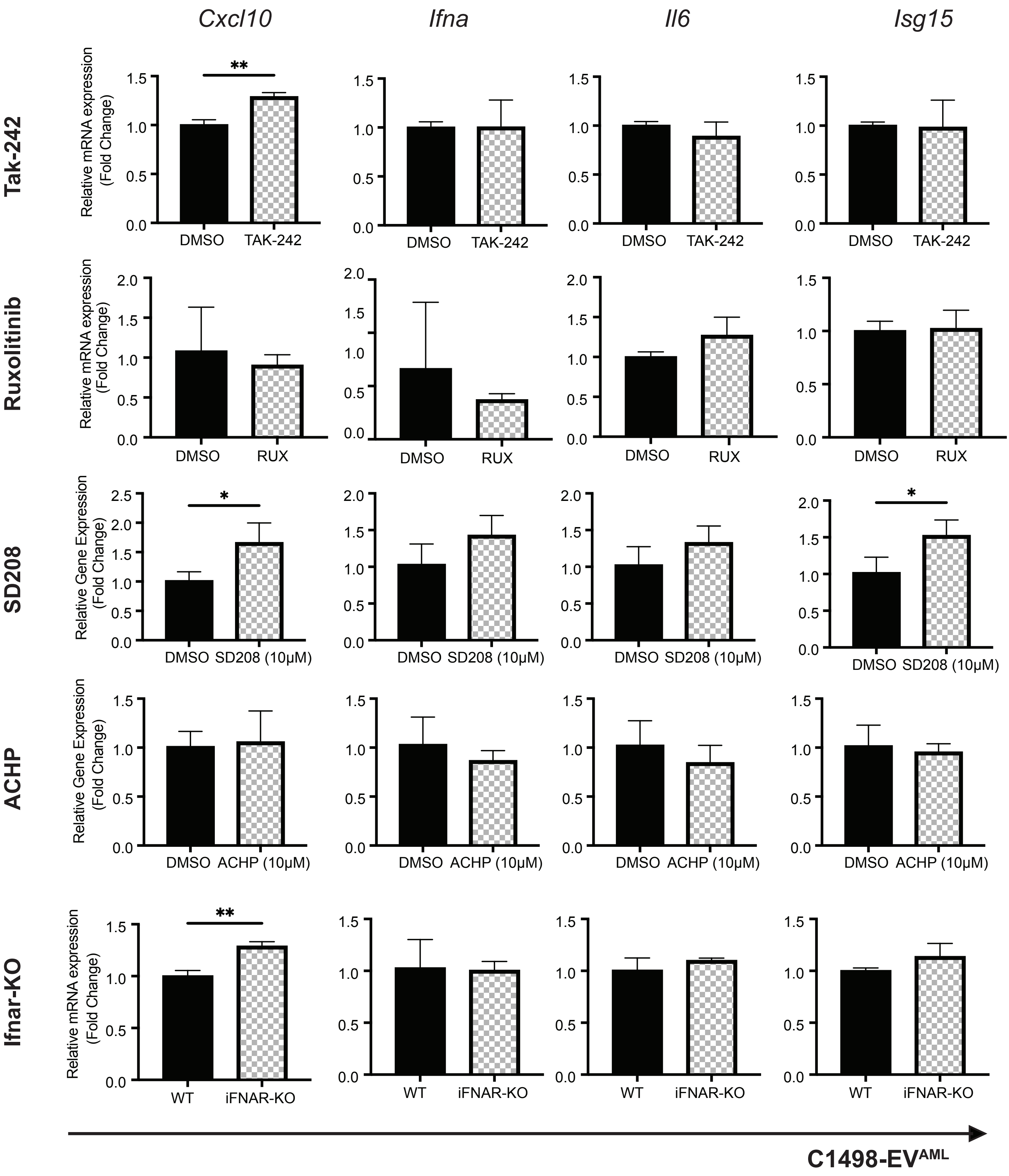

Supplementary Figure 7 - Additional inhibition study on C1498-EV^{AML} mediated inflammation in HSPCs. FACS-sorted HSPCs were pretreated with either TAK2-42, ruxolitinib, SD208, or ACHP prior to C1498-EV^{AML} challenge. To test the involvment of interferon alpha receptor, we subjected FACS-sorted IFNAR-KO HSPCs to C1498-EV^{AML} challenge. (n=3) Values expressed as mean±s.d., Statistical significance calculated using Student's t-test. * $p<0.05$; ** $p<0.01$.

Supplementary Figure 8

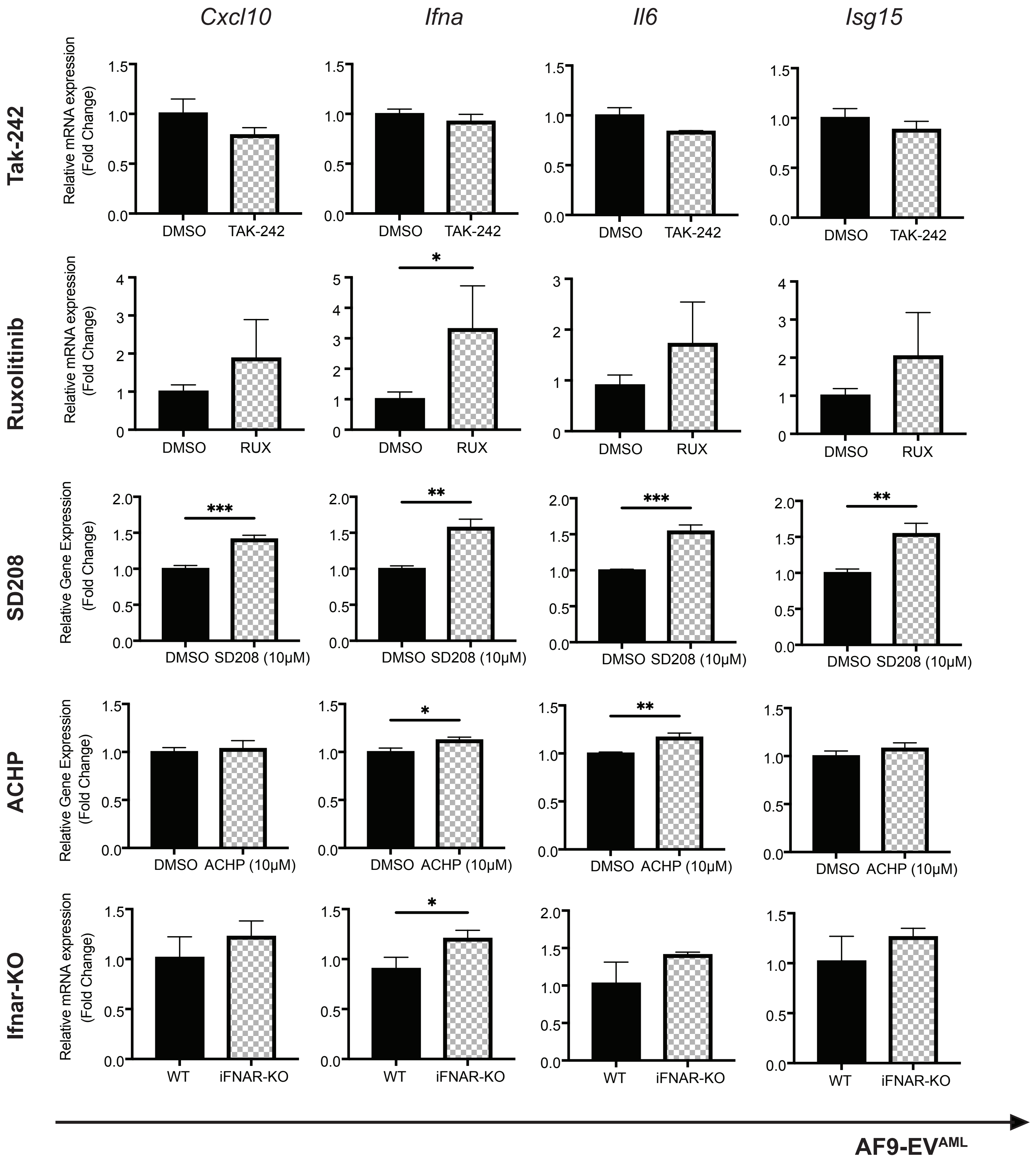

Supplementary Figure 8 - Additional inhibition study on AF9-EV^{AML} mediated inflammation in HSPCs. FACS-sorted HSPCs were pretreated with either TAK2-42, ruxolitinib, SD208, or ACHP prior to AF9-EV^{AML} challenge. To test the involvment of interferon alpha receptor, we subjected FACS-sorted IFNAR-KO HSPCs to AF9-EV^{AML} challenge (n=3). Values expressed as mean±s.d., Statistical significance calculated using Student's t-test. * p<0.05; ** p<0.01; *** p<0.001.

Supplementary Figure 9

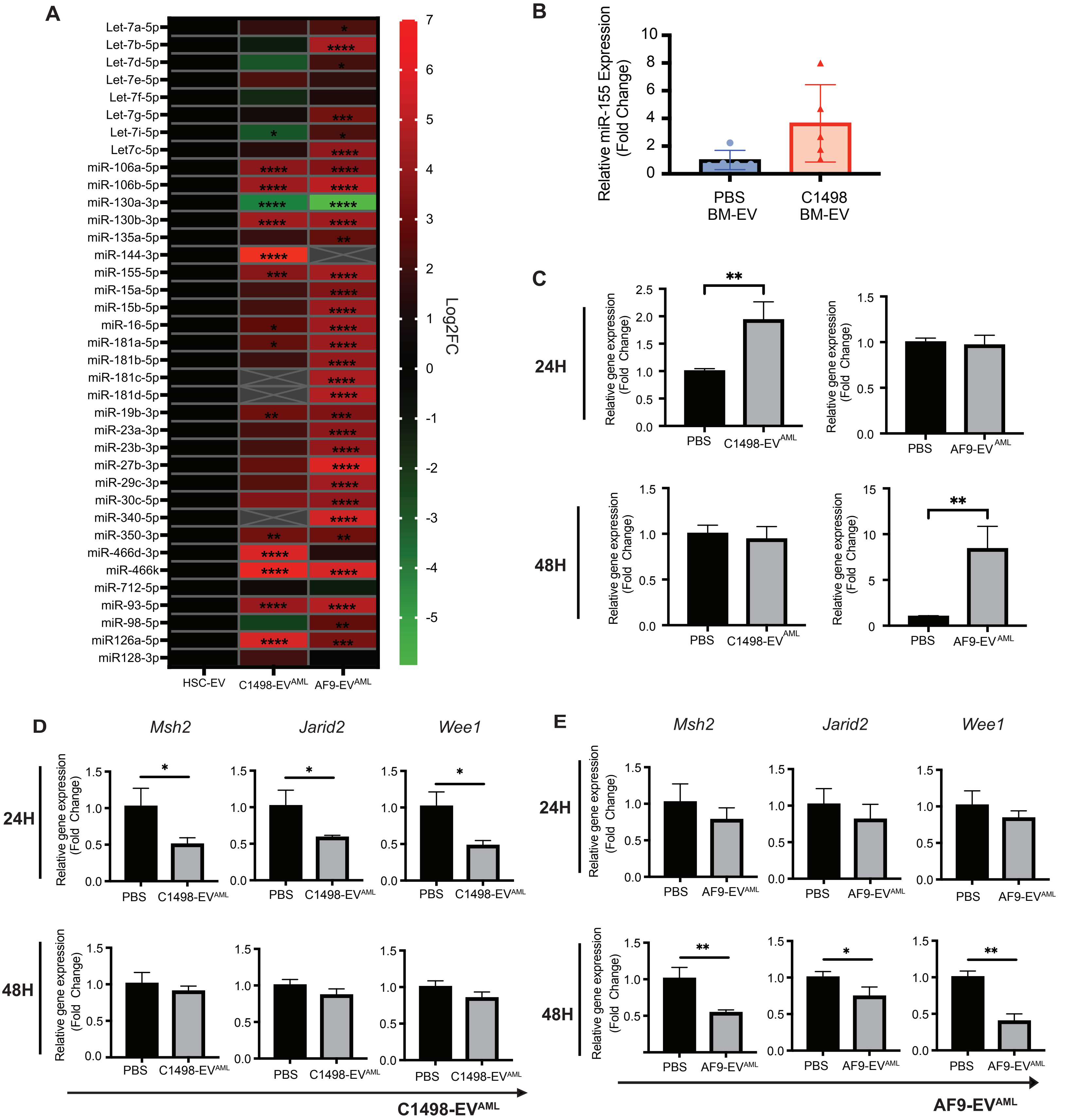

Supplementary Figure 9 - EV^{AML} miRNA cargo analysis. (A) miRNA expression profile show enriched inflammation-related miRNAs in C1498-EV^{AML} (n=2) and AF9-EV^{AML} (n=3) cargo compared to HSC-derived EVs (n=3). Expressions are normalized to U6. (B) Enrichment of miR-155 in the BM-EV of C1498-engrafted mice (n=5). (C) Elevated miR-155 expression in C1498-EV^{AML} and AF9-EV^{AML}-challenged HSPCs at 24 and 48 hours. Gene expression analysis of HSPCs for miR-155 targets (D) 24 hours and (E) 48 hours post EV^{AML} challenge (n=3). Values expressed as mean±s.d., Statistical significance calculated using Student's t-test. * $p<0.05$; ** $p<0.01$.

Supplementary Figure 10

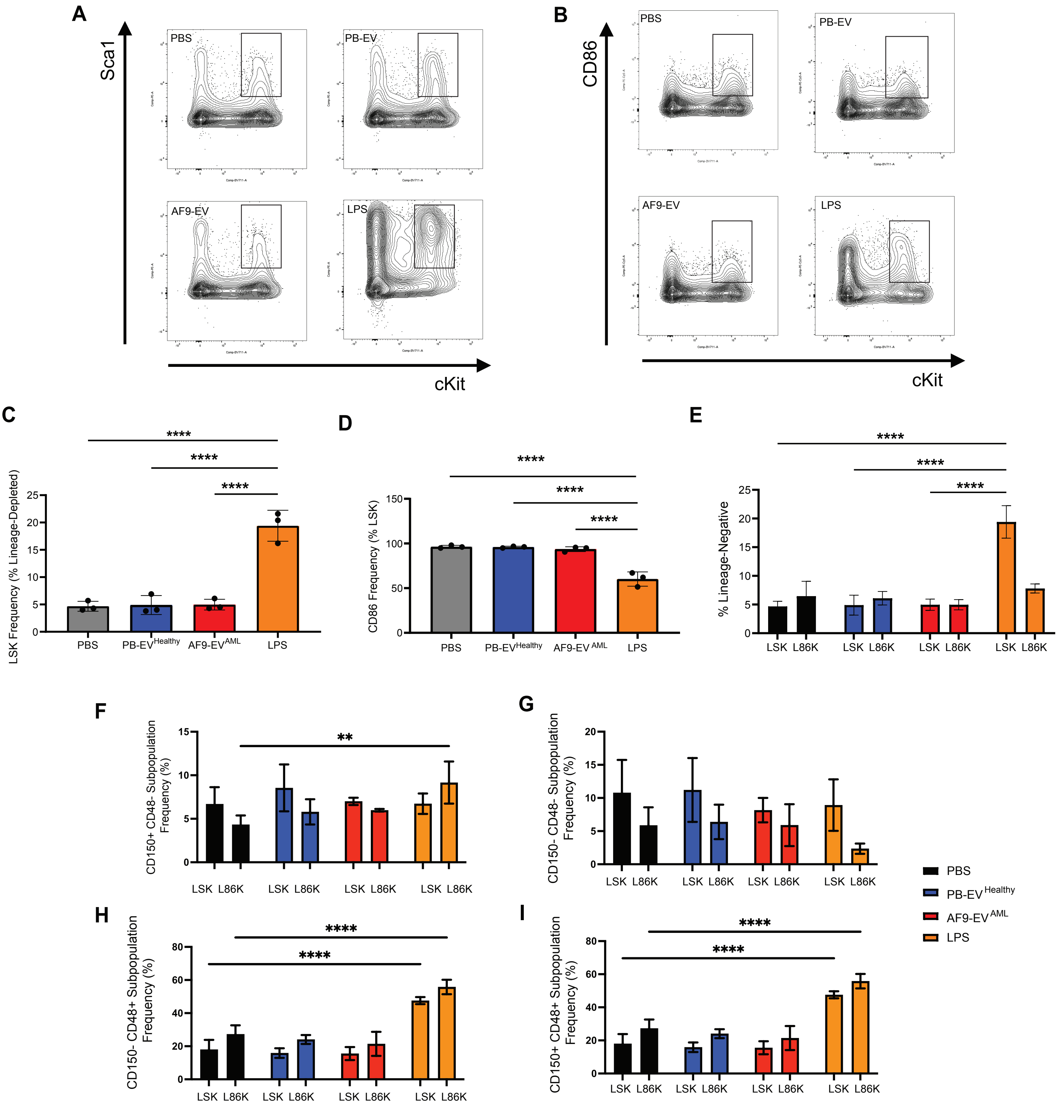

Supplementary Figure 10 – Analysis of LSK and L86K frequency following PBS, PB-EV^{Healthy}, AF9-EV^{AML}, and LPS challenge. Representative LSK (A) and L86K (B) flow cytometery gating. LSK Frequency (C), CD86 Frequency in LSK (D), comparative LSK and L86K frequency (E). HSPC subpopulation analysis in LSK and L86K fractions for: CD150+ CD48- population (F), CD150-CD48- population (G), CD150-CD48+ population (H), and CD150+CD48+ population (I). Values expressed as mean±s.d., Statistical significance calculated using ANOVA. * $p<0.05$; ** $p<0.01$; *** $p<0.001$; **** $p<0.0001$.

Supplementary Table 1 – List of Antibodies and Reagents Used

Name	Manufacturer	Catalogue Number
**Antibody**		
Brilliant Violet 421^{TM} anti-mouse CD45.1 Antibody (clone: A20)	BioLegend	110732
Brilliant Violet 650^{TM} anti-mouse CD45.2 Antibody (clone: 104)	BioLegend	109836
FITC anti-mouse CD3a Antibody (clone: 500A2)	BioLegend	152304
FITC anti-mouse CD11b Antibody (clone: M1/70)	BioLegend	101206
FITC anti-mouse CD5 Antibody (clone: 53-7.3)	BioLegend	100606
FITC anti-mouse CD4 Antibody (clone: Gk1.5)	BioLegend	100406
FITC anti-mouse Gr-1 Antibody (clone: R86-8C5)	BioLegend	108406
FITC anti-mouse B220 Antibody (clone: RA3-6B2)	BioLegend	103206
PE anti-human CD271 (NGFR) Antibody (clone: ME20.4)	BioLegend	345106
PE anti-mouse CD117-PE Antibody (clone: 2B8)	BioLegend	105808
APC Anti-mouse CD48 Antibody (clone: HM48-1)	BioLegend	103412
APC/Cyanine7 anti-mouse Sca-1 Antibody (clone:D7)	BioLegend	108126
PE/Cyanine 7 anti-mouse CD150 Antibody (clone: TC15-12F12.2)	BioLegend	115914
PE/Cyanine 5 anti-mouse CD135 Antibody (clone: A2F10)	BioLegend	135312
**Medium / Cytokines**		
Dulbecco's Modified Eagle's Medium	Corning	10-013-CM
RPMI 1640 Medium	Gibco	11875085
BenchMark Fetal Bovine Serum	GeminiBio	100-106
Penicillin/Streptomycin	Gibco	10378016
Doxycycline	Sigma	D9891
StemSpan SFEM	STEMCELL Technologies	09650
StemSpan SFEM II	STEMCELL Technologies	09655
Recombinant Murine Tpo	Peprotech	315-14
Recombinant Murine Flt3-l	Peprotech	250-31L
Recombinant Murine Scf	Peprotech	250-03
Recombinant Murine Il-3	Peprotech	213-13

Recombinant Murine Il-6	Peprotech	216-16
Recombinant Human IL-6	Peprotech	200-06
Recombinant Human FLT3-L	Peprotech	300-19
Recombinant Human SCF	Peprotech	300-07
Recombinant Human TPO	Peprotech	300-18
StemRegenin1	STEMCELL Technologies	72344
U171	APExBIO Technology	A8950
**Reagents / Kits**		
ACHP	Bio-Techne	4547
SD208	Bio-Techne	3269
JQ-1	MedChemExpress	HY-13030
Actinomycin D	Gibco	11805017
Rapamycin	Alfa Aesar	J62473
EasySep™ Mouse Hematopoietic Progenitor Cell Isolation Kit	STEMCELL Technologies	19856
RNeasy Plus Micro Kit	Qiagen	74034
miRNeasy Mini Kit	Qiagen	217084
SuperScript™ IV VILO™ Master Mix	Invitrogen	11756050
PowerUp™ SYBR™ Green Master Mix for qPCR	Applied Biosystems	A25742
TaqMan™ Fast Advanced Master Mix for qPCR	Applied Biosystems	4444557
TaqMan™ MicroRNA Reverse Transcription Kit	Applied Biosystems	4366596
RT² Profiler™ PCR Array Human Inflammatory Response & Autoimmunity (GeneGlobe ID: PAHS-077ZE-4)	Qiagen	330231
RT² First Strand Kit	Qiagen	330401
RT² SYBR Green ROX qPCR Mastermix	Qiagen	330521
Mouse Inflammatory Response & Autoimmunity Focus V2, miRCURY LNA miRNA Focus PCR Panel (GeneGlobe ID: YAMM-205YE-2)	Qiagen	339325
miRCURY LNA RT Kit	Qiagen	339340
miRCURY LNA SYBR Green PCR Kit	Qiagen	339345

Supplementary Table 2 - Sequences for quantitative PCR analysis primers

Gene	Species	Sequence Direction	Sequence
Isg15	Mus musculus	Forward	GGAACGAAAGGGGCCACAGCA
	Mus musculus	Reverse	CCTCCATGGGCCTTCCCTCGA
Cxcl10	Mus musculus	Forward	CCAAGTGCTGCCGTCATTTTC
	Mus musculus	Reverse	GGCTCGCAGGGATGATTTCAA
Gapdh	Mus musculus	Forward	AGGTCGGTGTGAACGGATTTG
	Mus musculus	Reverse	TGTAGACCATGTAGTTGAGGTCA
IFN-alpha	Mus musculus	Forward	CCTGAGAGAGAAGAAACACAGCC
	Mus musculus	Reverse	TCTGCTCTGACCACYTCCCAG
Il-6	Mus musculus	Forward	CCAAGAGGTGAGTGCTTCCC
	Mus musculus	Reverse	CTGTTGTTCAGACTCTCTCCCT
