# Supplementary material for: Inflammatory recruitment of healthy hematopoietic stem and progenitor cells in the acute myeloid leukemia niche
Source: Leukemia. 2024 Jan 16;38(4):741–50. doi: 10.1038/s41375-024-02136-7 (PMC10997516; doi:10.1038/s41375-024-02136-7)
Supplement: Supplementary file 2 — Supplementary Expanded Methods [file 41375_2024_2136_MOESM2_ESM.pdf]

SUPPLEMENATRY – EXPANDED METHODS

Cell Culture

Murine AML cell culture: C1498 cells (also known as TiB-49) were obtained from Dan Marks (Oregon Health & Science University). C1498 cells were cultured in Dulbecco's Modified Eagle Medium (DMEM) (Corning, Corning, NY, USA) supplemented with 10% fetal bovine serum (FBS; Mycoplasma were tested regularly and Benchmark, GeminiBio, West Sacramento, CA, USA) and penicillin/streptomycin (Pen/Strep;100U/mL, Gibco) and cultured cells in humidified incubator chamber at 37*°C*/5% CO_2. *iMLL-AF9 blasts* were flushed and FACS-sorted (NGFR$^+$ cKit$^+$ Gr-1$^+$ Mac-1$^+$; as previously described [29]) from BM of fully DOX-induced homozygous iMLL-AF9 mice, and were cultured ex v*ivo* in complete StemSpan SFEM medium (STEMCELL Technologies, Vancouver, Canada) composed of Tpo (50ng/mL; Peprotech), Flt3 ligand (50ng/mL; Peprotech), Scf (100ng/mL; Peprotech), and DOX (2μg/L; Sigma; St. Louis, MO, USA). Human AML cell culture: MOLM-14 and U-937 cells were cultured in Roswell Park Memorial Institute (RPMI)-1640 medium (Gibco) supplemented in 10% FBS (GeminiBio) and Pen/Strep (100U/mL, Gibco). HL-60 cells were cultured in RPMI-1640 medium supplemented in 20% FBS (GeminiBio) and Pen/Strep (100U/mL, Gibco). Cell lines were tested and negative for mycoplasma. Cells were seeded HSPCs (Lin$^-$ cKit$^+$ Sca1$^+$) were FACS-sorted and cultured in liquid culture using StemSpan (STEMCELL Technologies) supplemented with Il-3 (10ng/mL; Peprotech,Cranbury, NJ, USA), Il-6 (10ng/mL; Peprotech), Scf (50ng/mL; Peprotech), and PenStrep (1%). Human BM CD34+ cells were purchased from ATCC and cultured in StemSpan SFEM II medium (STEMCELL Technologies) supplemented with IL-6 (100 ng/mL; Preprotech), Flt3 ligand (100 ng/mL; Peprotech), SCF (100 ng/mL; Peprotech), TPO (100 ng/mL; Peprotech), StemRegenin1 (1μM; STEMCELL Technologies), U171 (35 nM; APExBIO Technology) and Pen/Strep (100U/mL). Inhibition studies were carried out by first treating HSPC with inhibitors, followed by EV^{AML} challenge. Inhibitor concentration and pretreatment durations are as follow: actinomycin D (2μg/mL; 4hrs; Gibco); JQ1 (1μM; 3 hrs; MedChemExpress, Monmouth Junction, NJ, USA); rapamycin (1μM; 5 hrs; Alfa Aesar, Haverhill, MA, USA); ACHP (10μM; 3hrs; Bio-Techne, Minneapolis, MN, USA), SD208 (10μM, 3hrs, Bio-Techne),. Following pretreatment, HSPCs were challenged with C1498- and AF9- EV^{AML} and cells were harvested for gene expression analysis 2 hours following EV^{AML} exposure.

Animal Models

Wildtype C57BL/6J-CD45.1 (B6.SJL-$Ptprc^a$ $Pepc^b$/BoyJ) mice were purchased from The Jackson Laboratory. iMLL-AF9 ((NOD.Cg-Kit $^{W-41J}$ Tyr $^+$ $Prkdc^{scid}$ $Il2rg^{tm1Wjl}$/ThomJ) were generously gifted by Dr. Shangqin Guo (Yale School of Medicine, Yale University). B6 CD45.1/2 were generated by crossing WT C57BL/6J with B6 CD45.1 mice for experiments. C1498 AML mice were generated by injecting C1498 cells (1E6 cells) into non-conditioned CD45.1 recipients (8-12 weeks; female) via tail vein. *iMLL-AF9 chimeric mice* were generated by co-transplanting BM harvested from homozygous iMLL-AF9 mice (CD45.1; 5E5 cells) and WT B6 mice (CD45.1/2; 5E5 cells) into lethally irradiated C57BL/6J (CD45.2) recipients (8-12 weeks; female) via tail vein. AML leukemogenesis was initiated via doxycycline (DOX) water feed (supplemented with 1g/L DOX and 10g/L sucrose) 4-8 weeks post BM transplantation. *Serial in vivo EV injection experiments* were performed by injecting either AF9-EV^{AML} (1-2E9 particles per injection) or PB-EV (1-2E9 particles per injection) into B6 CD45.1 mice (8-12 weeks; female) every 24 hours for 3 days via intravenous (for the first two injections) and intraosseous (third injection). Either PBS or lipopolysaccharides (LPS; 3mg/kg; 1 dose intraperitoneal) were injected as controls. All animal studies were conducted with a N of three at minimum. All animal experiments were conducted in accordance with Institutional Animal Care and User Committee (IACUC) protocol at the Children's Hospital of Philadelphia.

Transcriptional gene expression assays

RNA from FACS-sorted HSPCs were extracted using RNEasy Micro Plus Kit (Qiagen, Hilden, Germany) according to manufacturer's protocol. *qRT-PCR:* Reverse transcription were performed using SuperScript IV RT (Thermo Fisher Scientific, Waltham, MA, USA) and qRT-PCR using appropriate primers (Supplementary Table 1). *RNA-Seq:* Libraries were generated using NEX Rapid Dir RNA-Seq 2.0 Kit (Perkin Elmer) and sequenced using NovaSeq (Illumina, San Diego, CA, USA). RNA-seq data were preprocessed using the nf-core/RNAseq pipeline version 3.9. In brief, FastQC version 0.11.9 was used to assess the quality of 100 bp paired-end reads. Trim Galore! version 0.6.7 was then used to trim adapters and low quality bases from raw reads. Reads were aligned to GRCm38 (mm10) using STAR version 2.7.10a and quantified using RSEM version 1.3.1. Duplicates were removed using picard MarkDuplicates version 2.27.4-snapshot. Following preprocessing, data were filtered using the R package WGCNA version 1.69 to remove genes with many missing entries or zero variance. Differential expression between pairs of groups was estimated using DESeq2 version 1.38.3. Genes were considered to have significant differential expression if there was an adjusted p-value of less than 0.05. Pathway enrichment

analysis was performed using genes with significant differential expression between each group pairing ranked by their log2foldchange using the R package fGSEA version 1.24.0. *scRNA-Seq:* Gel-in-beads and libraries were generated using Chromium Next GEM Single Cell 3' Reagent Kits v3.1 (10x Genomics) and sequenced using NovaSeq (Illumina). Cell Ranger 7.0.1. was used to map the sequencing data. The raw sequencing data (FASTQ files) was mapped to the mouse reference genome 2020-A (GRCm38, GENCODE vM23/Ensembl 98). Data from all samples were loaded in R (version 4.2.2) and processed using the Seurat package (version 4.3.0 – Hao et al. Cell 2021). Cells with low UMI counts were filtered out. The filtering thresholds were determined independently for each library to exclude outliers (A1: 1800 UMIs/cell; A2: 3000 UMIs/cell; A3: 1800 UMIs/cell; B1: 2000 UMIs/cell; B3: 2000 UMIs/cell). Cells with more than 10% mitochondrial genes were also removed. Cells from library B2 were excluded due to the low quality of the library. The dataset was normalized for sequencing depth per cell and log-transformed using a scaling factor of 10,000. Principal component analysis (PCA) was performed on the 2,000 most variable features. The first 29 PCs were used to generate the uniform manifold approximation and projection (UMAP). Cell cycle was scored using the CellCycleScoring function of Seurat. Inspection of the UMAP revealed differential distribution assigned to cell cycle. Cell cycle effects were then regressed out using the vars.to.regress parameter in Seurat's ScaleData function. After regression, principal component analysis (PCA) was performed again on the 2,000 most variable features, and the first 27 PCs were used for dimensionality reduction and clustering. Batch effects between libraries were removed using the Harmony package (version 0.1.1). Clustering was performed using the Louvain algorithm, based on a shared nearest neighbour graph, as implemented in the FindClusters function in Seurat with parameters k.param = 5 and resolution = 0.3. The differentially expressed genes per cluster were calculated using the Wilcoxon rank sum test as implemented in the FindAllMarkers function in Seurat, with parameters logfc.threshold = 0.1, return.thresh = 0.01, only.pos = TRUE, min.pct = 0.1. Differentially expressed genes between conditions (AML vs PBS) were calculated using the Wilcoxon rank sum test as implemented in the FindAllMarkers function in Seurat with parameters logfc.threshold = 0.1, return.thresh = 0.01 and min.pct = 0.1. We then performed an over- representation analysis of the significantly differentially expressed genes using the enricher function in the clusterProfiler package (version 4.6.2) with the collections "MH: hallmark gene sets", "M2: curated gene sets", "M5: ontology gene sets" and "M8: cell type signature gene sets" from the Mouse Molecular Signatures Database. Cells were annotated using gene signatures for HSPCs subpopulations (HSCs, MPP-2, MPP-3, and MPP-4) reported by Pietras et al (26). The gene signature for each population was defined by the top 100 genes ranked by the Significance Analysis of Microarrays (SAM) scores. We

calculated a module score for each cell based on the average expression levels of every gene signature, subtracted by the aggregated expression of randomly selected control genes. Each cell was assigned to the population with the highest positive score. Cells with only negative scores were annotated as “Unclassified”. Differentially expressed genes between conditions (AML vs PBS) per cell type were calculated using the Wilcoxon rank sum test. Over-representation analysis of the differentially expressed genes was performed using the enricher function in the clusterProfiler package (version 4.6.2) with the collections “MH: hallmark gene sets”, “M2: curated gene sets”, “M5: ontology gene sets” and “M8: cell type signature gene sets” from the Mouse Molecular Signatures Database. Gene expression profiling assay: Extracted RNA were subjected to gene expression analysis using RT2 Profiler PCR Array Mouse Inflammatory Response & Autoimmunity Panel according to manufacture’s recommended protocol. miRNA extraction: miRNA were extracted from either cells or EVs using miRNeasy Mini Kit (Qiagen) according manufacture’s protocol. For selected miRNA targeted analysis, miRNA were first subjected to RT using TaqMan MicroRNA Reverse Transcription Kit (Thermo), followed by real-time PCR using TaqMan Advanced miRNA Assays (Thermo). For miRNA panel profiling, extracted miRNA were assayed using *“Mouse Inflammatory Response & Autoimmunity Focus V2, miRCURY LNA miRNA Focus PCR Panel”* (Qiagen) according to manufacture’s protocol.

FACS cell sorting or analysis

Cells were sorted and analyzed using either FACSAria Fusion cell sorter or FACS Canto II (Becton Dickinson, Franklin Lakes, NJ, USA). Lineage-negative cells were enriched using EasySep Mouse Hematopoietic Progenitor Cell Isolation Kit (Stem Cell Technologies) according to manufacturer’s protocol prior to sorting. Cells were stained with appropriate mouse antibodies (Supplementary Table 2) for LT-HSC (Lin^- $cKit^+$ $Sca\text{-}1^+$ $CD150^+$ $CD48^-$), ST-HSC (Lin^- $cKit^+$ $Sca\text{-}1^+$ $CD150^-$ $CD48^-$), MPP-2 (Lin^- $cKit^+$ $Sca\text{-}1^+$ $CD150^+$ $CD48^+$), and MPP-3/4 (Lin^- $cKit^+$ $Sca\text{-}1^+$ $CD150^-$ $CD48^+$) analysis.

Extracellular vesicles isolation and characterization

For EV^{AML} generation, EV-depleted FBS were used instead of regular FBS to make complete DMEM and RPMI medium. EV-depleted FBS were generated by subjecting 0.1μm-filtered FBS (GeminiBio) to ultracentrifugation at 100,000 x g for 20 hours at 4°C, where the supernatant fraction served as EV-depleted FBS. AML cells (7E6) were seeded and cultured for 72 hours, followed by EV^{AML} harvests from conditioned medium using differential centrifugation methods involving: 400 x g for 10 mins, 2,000 x g for 20 mins, 10,000 x g for 20 mins, and 100,000 x g for

2 hours. Ultracentrifugation were performed using either Optima L-60 or L-80 instrument with either SW-28, SW-40, or SW-55-Ti rotors (Beckman Coulter, Brea, CA, USA). *For BM-EVs and PB-EV*: BM plasma was obtained by flushing hind legs (tibia and femur) using HBSS buffer supplemented with Pen/Strep (100U/mL), followed by 400 x g centrifugation for 10 minutes at 4°*C*. PB plasma was obtained by subjecting PB to 400 x g centrifugation for 10 minutes at 4°*C. EVs from both BM and PB plasma were pelleted using* differential centrifugation methods described above. Pelleted EVs were resuspended in 0.1μm-filtered sterile PBS supplemented with 1% P/S. EVs were quantified utilizing tunable resistance pulse sensing method using qNano Gold (Izon, Christchurch, New Zealand), with either NP150 or NP200 nanopores.

BM plasma protein assays

Murine BM plasma fluids were collected by flushing 2 hind legs (femur and tibia) with Hanks' Balanced Salt Solution (HBSS) buffer in fixed volumes (10mL). Cells from flushed BM were pelleted using centrifugation, and the supernatant fraction were collected and considered as "BM plasma". BM plasma were directly subjected to protein quantification using Luminex-based multiplex assay mouse cytokine/chemokine 44-Plex Discovery Assay, which was performed by Eve Technologies (Calgary, Alberta, Canada). Human BM plasma samples from AML patients and healthy donors were obtained from Dr. Martin Carroll and Dr. Nicolas Skuli from the Stem Cell and Xenograft Core (Perelman School of Medicine, University of Pennsylvania). Human BM plasma samples were collected from BM aspirates following cell separation using Ficoll. The aqueous plasma layer on the top were subjected to protein quantification using enzyme-linked immunosorbent assay (ELISA) (Thermo Fisher Scientific).

Statistical analysis

Statistical analyses for data generated by qRT-PCR and flow cytometry were performed using GraphPad Prism software (GraphPad Software Inc, La Jolla, CA, USA). Statistical analysis methods used in RNA-Seq and scRNA-Seq analyses is reported in the "Transcriptional gene expression assay" section above. Statistical analysis was calculated by either one-way ANOVA with Tukey test, 2-way ANOVA with Dunnett correction, or two-tailed Student's t-tests where appropriate. P-values of less than 0.05 were considered statistical significance and denoted as * $p < 0.05$;** $p < 0.01$; *** $p < 0.001$; **** $p < 0.0001$. No tests were used in sample size estimation, and methods of randomization and blinding were utilized.

Graphical illustrations

Illustrations were generated utilizing a licensed application BioRender with rights to use in publications.
